# Evaluation of the Effect of Andrographolide on Atherosclerotic Rabbits Induced by *Porphyromonas gingivalis*

**DOI:** 10.1155/2014/724718

**Published:** 2014-08-18

**Authors:** Rami Al Batran, Fouad Al-Bayaty, Mazen M. Jamil Al-Obaidi, Saba F. Hussain, Tengku Z. Mulok

**Affiliations:** ^1^Center of Periodontology Studies, Faculty of Dentistry, Universiti Teknologi Mara (UiTM), 40450 Shah Alam, Selangor Darul Ehsan, Malaysia; ^2^Center of Paediatric Dentistry and Orthodontics Studies, Faculty of Dentistry, Universiti Teknologi MARA (UiTM), 40450 Shah Alam, Selangor Darul Ehsan, Malaysia; ^3^Center of Biomolecular Science, Faculty of Applied Science, Universiti Teknologi MARA (UiTM), 40450 Shah Alam, Selangor Darul Ehsan, Malaysia

## Abstract

Epidemiologic evidence has demonstrated significant associations between atherosclerosis and* Porphyromonas gingivalis* (*Pg*). We had investigated the effect of andrographolide (AND) on atherosclerosis induced by* Pg* in rabbits. For experimental purpose, we separated thirty male white New Zealand rabbits into 5 groups. Group 1 received standard food pellets; Groups 2–5 were orally challenged with* Pg*; Group 3 received atorvastatin (AV, 5 mg/kg), and Groups 4-5 received 10 and 20 mg/kg of AND, respectively, over 12 weeks. Groups treated with AND showed significant decrease in TC, TG, and LDL levels (*P* < 0.05) and significant increase in HDL level in the serum of rabbits. Furthermore, the treated groups (G3–G5) exhibited reductions in interleukins (IL-1*β* and IL-6) and C-reactive protein (CRP) as compared to atherogenicgroup (G2). The histological results showed that the thickening of atherosclerotic plaques were less significant in treated groups (G3–G5) compared with atherogenicgroup (G2). Also, alpha-smooth muscle actin (*α*-SMA) staining decreased within the plaques of atherogenicgroup (G2), while it was increased in treated groups (G3–G5). Lastly, groups treated with AV and AND (G3–G5) showed significant reduction of CD36 expression (*P* < 0.05) compared to atherogenicgroup (G2). These results substantially proved that AND contain antiatherogenic activity.

## 1. Introduction

Periodontal disease and heart disease are inflammatory conditions. Generally, it is considered that periodontal disease and heart disease are connected with each other; however, it has not been clear whether having periodontal disease leads to heart disease or vice versa. According to the journal of the American Medical Association [1], patients who have periodontal disease and at least one risk factor for heart disease should have a medical evaluation for heart problems. On the other hand, patients who have heart disease should regularly check for signs of periodontal disease.* Pg* is a nonmotile and rod-shaped anaerobic, gram-negative bacterium that can be found in the mouth of an individual. This bacterium is the principal cause of periodontal disease [2]. Besides causing infections to humans, the way that it operates is very unique; since it is a gram-negative bacterium, it can attach to the subgingival area of periodontal pocket and cause an inflammation of the periodontal tissue [3]. Recent epidemiological studies have demonstrated an association among periodontal disease, cardiovascular disease, and atherosclerosis [4]. An association between atherosclerosis and* Pg*, a major periodontopathogen, has been shown [5]. However, the question of whether this relationship is causal or coincidental still exists. Many individuals with evidence of atherosclerosis have demonstrated seropositivity to this pathogen. Recently, evidence from diverse sources has suggested that* Pg* can activate host innate immune responses, associated with atherosclerosis [6]. Various studies have confirmed that the inflammatory response to* Pg* could exacerbate vascular inflammation via secreted cytokines and/or chemokines, which ultimately develop atherosclerosis. Meanwhile, the cytokine and chemokines interact in the progression of atherosclerosis. Moreover, the reaction of endothelial cells in response to* Pg* and its various virulence factors are diverse, and the expression of chemokine differs through different signal transduction pathways, accordingly. Results from these studies have reinforced the connection between* Pg* and atherosclerosis, and the role of* Pg* in the initiation and progression of atherosclerosis has been presented [7].

A number of studies have reported the use of a large variety of medicinal plants for treating several ailments [8–12].* Andrographis paniculata* (Burm.f.) Nees (Acanthaceae) is a long-established therapeutic plant, commonly found in South East Asia and originated from India to Indo-China. The plant is generally known as “king of bitter.” The main bioactive compound is AND as the core dynamic code and other codes such as 14-deoxy-11, 12-didehydroandrographolide, and 14-deoxyandrographolide. Recent studies on AND have shown several pharmacological effects such as hypotensive [13], antihyperglycemic [14], accelerates wound healing [15], gastroprotective [16], antioxidant [11] and anticancer [17]. The present study has been aimed at assessing the antiatherogenic activity of AND in atherosclerotic rabbits.

## 2. Materials and Methods

### 2.1. Chemicals

For the purpose of this study, AND was purchased from Sigma Aldrich (USA) and* Pg* strain 33277 from ATCC (USA), whereas AV was acquired from the University Malaya Medical Centre (UMMC) Pharmacy and used as the reference drug at a dose of 5 mg/kg body weight.

### 2.2. Culturing Bacteria

Under anaerobic condition,* Pg* ATCC strain 33277 was cultured on anaerobic blood agar plates (Becton Dickinson Co.) in an aerobic chamber (Coy Laboratory Products Inc.) with 85% N_2_, 5% H_2_, and 10% CO_2_, for 3 to 5 days, and then inoculated into Schaedler broth (Difco Laboratories), containing hemin and menadione for 24 hours, according to the previous protocol [7], with some modifications.

### 2.3. Animal Ethics

Thirty healthy male white New Zealand rabbits (3–3.5 kg) were acquired from the Animal House, University Technology Mara, and the experiment was approved by the Committee of Faculty of Dentistry, University Technology Mara, Ethic number [28/05/2013. 600-FF (PT. 5/2)]. The experimental rabbits were placed in separate cages and maintained on a twelve-hour day/night cycle at an ambient temperature, with* ad libitum* access to food and water.

### 2.4. Maximum Tolerated Dose (MTD)

Sixteen rabbits were used in this experiment and were divided into two groups, comprising eight male and eight female rabbits. All rabbits had free access to water and given normal diet. All animals were clinically examined on daily basis during the experiments. AND was diluted with water and orally administered to different groups at doses of 50, 100, and 500 mg/kg body weight, daily for four weeks. During the course of experiment we had observed and recorded any possible changes on physical appearance, body weight, and mortality.

### 2.5. Animal Grouping

Rabbits were divided into the following 5 groups (*N* = 6 per group).

*Group 1 (normal control)*. Rabbits were continuously fed with the standard pellets for 12 weeks. 

*Group 2 (atherogenic control).* Rabbits were continuously challenged orally with* Pg *ATCC 33277 (0.2 mL of 1.5 × 10^*x*12^ bacterial cells/mL in 2% CMC with PBS) five times a week for 12 weeks. 

*Group 3 (atherogenic + AV).* Rabbits were continuously challenged orally with* Pg *ATCC 33277 (0.2 mL of 1.5 × 10^*x*12^ bacterial cells/mL in 2% CMC with PBS) five times a week and continuously fed daily with AV (5 mg/kg) for 12 weeks. 

*Group 4 (atherogenic + AND LD).* Rabbits were continuously challenged orally with* Pg *ATCC 33277 (0.2 mL of 1.5 × 10^*x*12^ bacterial cells/mL in 2% CMC with PBS) five times a week and continuously fed daily with AND low dose (10 mg/kg) for 12 weeks. 

*Group 5 (atherogenic + AND HD).* Rabbits were continuously challenged orally with* Pg* ATCC 33277 (0.2 mL of 1.5 × 10^*x*12^ bacterial cells/mL in 2% CMC with PBS) five times a week and continuously fed daily with AND high dose (20 mg/kg) for 12 weeks.

### 2.6. Biochemical Measurement

The experimental rabbits were kept under fasting condition for at least 12 hours before blood sampling, to allow the relevant estimation of lipid profile levels. Blood was taken from the ear vein of nonanaesthetized rabbit into an open plain tube and centrifuged at 3500 g for 15 min to obtain serum. The levels of total cholesterol (TC), triglycerides (TG), low-density lipoprotein-cholesterol (LDL), high-density lipoprotein-cholesterol (HDL), and C-reactive protein (CRP) were analyzed from the serum of rabbits at the University of Malaya Medical Centre to evaluate the changes in biomarkers, using an automatic biochemistry analyzer (Dimension AR, DuPont, USA).

### 2.7. Proinflammatory Cytokines Measurement

IL-1*β* and IL-6 concentrations were measured in serum, using ELISA (enzyme-linked immunosorbent assay) (Abcam, USA) with sensitivity of 1 pg/mL and without cross reactivity against other cytokines, according to the manufacturer's recommendations.

### 2.8. Histopathological Study of Aorta

Aorta was harvested from the rabbits and placed in formaldehyde 10%. 24 h later, aorta was embedded in paraffin, cut at 4 *μ*m, stained with hematoxylin and eosin (H&E), and then scanned to assess pathological changes.

### 2.9. Immunohistochemistry Study

Shortly, 4-*μ*m sections of aortic tissue blocks were placed on poly-L-lysine coated slides for the purpose of performing immunohistochemistry. The slide sections were immersed in target retrieval solution (DAKO Lot 10069393), heated in microwave oven at 98°C for 20 minutes (maximum power 700 W), and then cooled at room temperature; anti-alpha smooth muscle actin antibody (abcam, ab7817), 1/50 dilution was used as primary antibody as previously described [18]. Briefly, the sections were incubated with each primary antibody as mentioned above for 1 hour; after rinsing thrice in Dako wash buffer (TBS), they were incubated with biotinylated secondary antibody, by following kit (LSAB system2-HRP) (Lot 10069908) for 1 hour at room temperature. After TBS rinses, the sections were incubated with streptavidin-horseradish peroxidase conjugate for additional 30 minutes at room temperature (DAKO), followed by a course of incubation in diaminobenzidine (DAB) DAKO (lot 10067468). Control immunohistochemistry reactions were performed to evaluate the specificity of the labels, omitting the primary antibody. Staining with hematoxylin was performed and used as a reference of the cytoarchitecture of the tissue.

### 2.10. Morphometric Analysis

Images were captured using a Nikon microscope (Y-THS, Japan) by two experienced observers in an independent and blinded fashion with the atherogenic control and treatment groups. The mean percentage of thickening of the intimal layer, foam cells, and density areas of alpha-SMA stain cell was microscopically assessed. Several sections were microscopically analyzed under high power fields (HPF) for each experimental group, using an optical image analyzer (ImagePro Plus 4.5, Media Cybernetics, Silver Spring, MD).

### 2.11. Western Blotting

Homogenized samples from the aorta were separated on 4–20% sodium dodecyl sulphate (SDS-PAGE) gels and the proteins were transferred to polyvinylidene fluoride (PVDF) membrane. Briefly, the membranes were blocked with 5% nonfat milk followed by primary antibodies, recognizing anti-CD36 antibody (ab78054) 1/1000 dilution, and incubated at 4°C overnight. The membranes were washed and incubated with HRP-conjugated goat anti-rabbit IgG. The densities were normalized to the total amount of protein loaded in each well, as determined by densitometry analysis of PVDF membranes, and stained with Amersham ECL Prime Western Blotting Detection Reagent (GE Healthcare). The proteins were visualized by chemiluminescence (UVP, Bio Spectrum, USA) and the densities of specific bands were quantitated by densitometry using (Image J 1.37 software, NIH, USA) [19]. Housekeeping protein *β*-actin (1 : 1000) was used as loading control.

### 2.12. Statistical Analysis

All values were reported as mean ± S.E.M. Data were analyzed by one-way ANOVA and Tukey's post hoc test for multiple comparison using SPSS 18 (SPSS Inc., Chicago, Illinois, USA). Significance was defined as ∗*P* < 0.05 compared to atherogenic group (G2).

## 3. Results

### 3.1. Maximum Tolerated Dose (MTD) of AND

The results presented in supplementary data (see Tables S1 and S2 in Supplementary Material available online at http://dx.doi.org/10.1155/2014/724718) show maximum tolerated dose of AND in male and female rabbits. Toxicology was assessed on the mortality rate. The dosages of AND at 50, 100, and 500 mg/kg body weight did not produce any mortality to the rabbits during four weeks.

### 3.2. Effect of AND on Lipid Profile

Table 1 shows the level of lipid parameters in serum among different experimental groups. Treated groups (G3–G5) showed a significant reduction of TG level (*P* < 0.05), as against atherogenic group (G2) at a value of 1.31 ± 0.01 mmol/L. Meanwhile, atherogenic group (G2) had higher level of TG, compared to normal control group (G1). AV group (G3) had the most significant reduction of TG level, followed by AND HD and AND LD. Atherogenic group (G2) had higher level of TC, compared to normal control group (G1). The total cholesterol level (TC) value for AV group (G3) was 15.21 ± 0.12 mmol/L which was the most significant reduction of TC level (*P* < 0.05), followed by AND HD group (G5) at value of 16.20 ± 0.18 mmol/L, compared to atherogenic group (G2) at value of 26.21 ± 0.29 mmol/L. Furthermore, AND LD (G4) had significantly reduced the TC level, compared to atherogenic group (G2); however, it was less effective than AV and AND HD (G3 and G5).

The LDL level was significantly lower (*P* < 0.05) in the AV and AND HD groups (G3, G5) at values of 13.65 ± 0.16 mmol/L and 14.37 ± 0.22 mmol/L, compared with the atherogenic group (G2). Atherogenic group (G2) had higher level of LDL, compared to normal control group (G1). The LDL level for AND LD (G4) was significantly decreased, compared to atherogenic group (G2). The HDL level in the atherogenic group (G2) was significantly decreased (*P* < 0.05) with a value of 0.44 ± 0.01 mmol/L, compared to normal control group (G1). Meanwhile, the AND HD group (G5) had significantly increased HDL level at value of 0.64 ± 0.01 mmol/L, compared to atherogenic group (G2). The AV group (G3) had a HDL level of 0.65 ± 0.01 mmol/L that was significantly higher than the atherogenic group (G2). Based on the lipid profile results, AV group (G3) had showed the best results, followed by AND HD (G5) and AND LD (G4), compared to atherogenic group (G2).

### 3.3. Effect of AND on C-Reactive Protein

**Figure 1:**
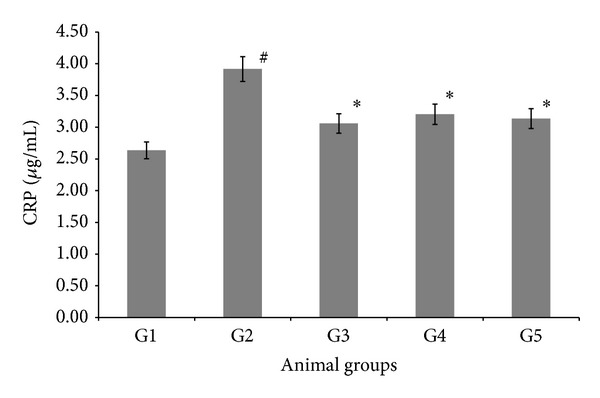
The effect of AND on C-reactive protein (CRP) in atherosclerotic rabbits. Group 1: normal control; Group 2: atherogenic control; Group 3: atherogenic + AV; Group 4: atherogenic + AND LD; Group 5: atherogenic + AND HD. Statistical analysis of the data was carried out using one-way analysis of variance (ANOVA) and Tukey's post hoc test for average comparison on SPSS 18.0. Mean values ± SEM (*N* = 6) were used. Significance difference was defined as *P < 0.05 compared to atherogenic group (G2); ^#^ *P* < 0.05 compared to normal control (G1).

C-reactive protein (CRP) level in serum was measured to determine the inflammation status of the experimental groups as shown in Figure 1. Generally, the atherogenic group (G2) had significantly higher levels of CRP (*P* < 0.05) at value 3.92 ± 0.09 (*μ*g/mL), compared with the normal group (G1). Outstandingly, the AV and AND HD groups (G3 and G5) had significantly lower levels of CRP (*P* < 0.05) at values 3.06 ± 0.03 (*μ*g/mL) and 3.14 ± 0.01 (*μ*g/mL), respectively, compared with the atherogenic group (G2). The CRP level for AND LD (G4) was significantly decreased, compared to atherogenic group (G2), but was at higher value than the AV and AND HD groups (G3 and G5).

### 3.4. Effect of AND on Proinflammatory Cytokines

**Figure 2:**
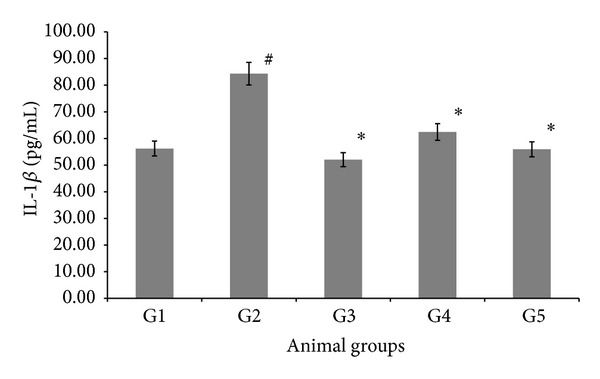
The effect of AND on interleukin 1 (IL-1*β*) in serum in atherosclerotic rabbits. Group 1: normal control; Group 2: atherogenic control; Group 3: atherogenic + AV; Group 4: atherogenic + AND LD; Group 5: atherogenic + AND HD. Statistical analysis of the data was carried out using one-way analysis of variance (ANOVA) and Tukey's post hoc test for average comparison on SPSS 18.0. Mean values ± SEM (*N* = 6) were used. Significance difference was defined as *P < 0.05 compared to atherogenic group (G2); ^#^ *P* < 0.05 compared to normal control (G1).

The effect of AND on interleukin 1 (IL-1*β*) activity in the different experimental groups is shown in Figure 2. Generally, rabbits in the atherogenic group (G2) had significantly higher (*P* < 0.05) levels of IL-1*β* at a value of 84.33 ± 0.67 (pg/mL), compared with the normal control group (G1). Notably, the experimental rabbits, treated with AND LD and HD (G4, G5) had significantly lower levels of IL-1*β*, at values of 62.45 ± 1.43 (pg/mL) and 55.95 ± 1.16 (pg/mL), respectively, compared to the atherogenic group (G2). Moreover, there were significant differences in the tested IL-1*β* level between AND HD (G5) and AV (G3), at a value of 52.07 ± 0.71 (pg/mL).

**Figure 3:**
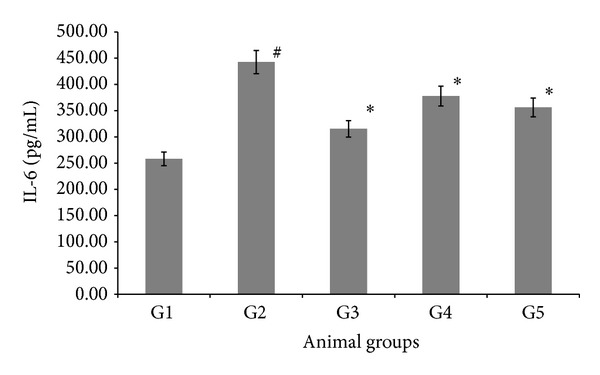
The effect of AND on interleukin 6 (IL-6) in serum in atherosclerotic rabbits. Group 1: normal control; Group 2: atherogenic control; Group 3: atherogenic + AV; Group 4: atherogenic + AND LD; Group 5: atherogenic + AND HD. Statistical analysis of the data was carried out using one-way analysis of variance (ANOVA) and Tukey's post hoc test for average comparison on SPSS 18.0. Mean values ± SEM (*N* = 6) were used. Significance difference was defined as *P < 0.05 compared to atherogenic group (G2); ^#^ *P* < 0.05 compared to normal control (G1).

The effect of AND on interleukin 1 (IL-6) activity among the different experimental groups is shown in Figure 3. Generally, the IL-6 results were similar to the IL-1*β* results. The IL-6 level in the atherogenic group (G2) was the highest at a value of 442.64 ± 0.75 (pg/mL) among the groups. However, the AV group (G3) had the lowest IL-6 levels at a value of 315.35 ± 0.78 (pg/mL), compared with the atherogenic group (G2). The AND HD group (G5) had significantly (*P* < 0.05) decreased IL-6 levels, compared to the atherogenic group (G2). Furthermore, AND LD (G4) had shown significantly decreased IL-6 levels (*P* < 0.05), compared to the atherogenic group (G2), but was at higher value than AV and AND HD (G3 and G5).

### 3.5. Histopathological Findings

**Figure 4:**
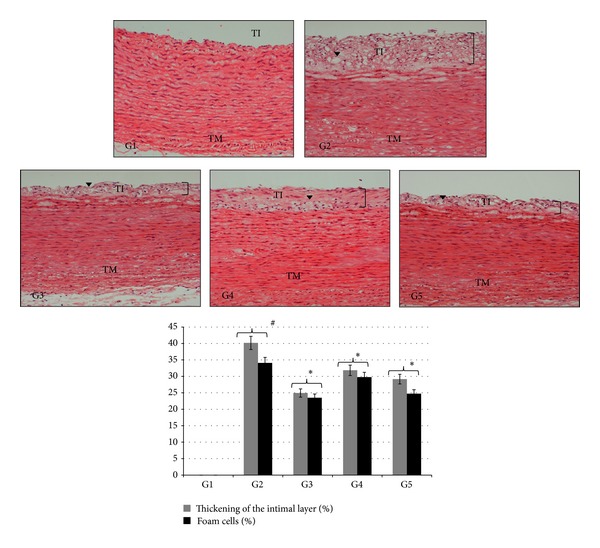
The effect of AND on histological sections of aorta in atherosclerotic rabbits (H&E). TI; tunica intima; TM; tunica media; arrowheads indicated the foam cells. Group 1: normal control; Group 2: atherogenic control; Group 3: atherogenic + AV; Group 4: atherogenic + AND LD; Group 5: atherogenic + AND HD. Image analysis was accomplished using an optical image analyzer (ImagePro Plus 4.5, Media Cybernetics, Silver Spring, MD). The data are expressed as percentage means ± SEM (*n* = 6) and were analysed using one-way analysis of variance (ANOVA) followed by Tukey's post hoc test for average comparisons on SPSS 18.0. Significance difference was defined as *P < 0.05 compared to atherogenic group (G2); ^#^ *P* < 0.05 compared to normal control (G1).

Histopathological examination of aorta sections from experimental groups is shown in Figure 4. Histological examination of the hematoxylin and eosin-stained section of aorta of normal group (G1) (rabbits fed with a normal diet) showed normal histological examination of aorta, with no aortic lesion or presence of foam cells, while the aorta examination of the atherogenic group (G2) showed thickening of the intimal layer and accumulation of lipids, leading to format foam cells, signaling the early phase in the development of atherosclerosis. The aorta of the groups treated with AV and AND (G3, G4, and G5) showed reduced accumulation of atherosclerotic lesion, due to decreased number of foam cells and cholesterol deposits in the aorta. The morphometric analysis of aorta section results had showed that the thickening of the intimal layer and foam cells was significantly increased (*P* < 0.05) in atherogenic group (G2), compared to normal control group (G1). Furthermore, treated groups (G3–G5) showed significantly decreased intimal layer and foam cells (*P* < 0.05), compared with atherogenic group (G2).

### 3.6. Immunohistochemistry Assessment

**Figure 5:**
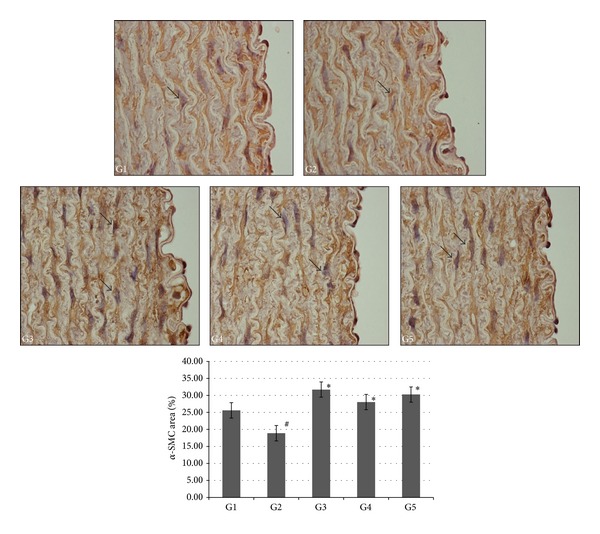
The effect of AND on *α*-smooth muscle actin of aorta in atherosclerotic rabbits. Arrows indicated were the positive staining of *α*-SMA. Group 1: normal control; Group 2: atherogenic control; Group 3: atherogenic + AV; Group 4: atherogenic + AND LD; Group 5: atherogenic + AND HD. Image analysis was accomplished using an optical image analyzer (ImagePro Plus 4.5, Media Cybernetics, Silver Spring, MD). The data are expressed as percentage means ± SEM (*n* = 6) and were analysed using one-way analysis of variance (ANOVA) followed by Tukey's post hoc test for average comparisons on SPSS 18.0. Significance difference was defined as *P < 0.05 compared to atherogenic group (G2); ^#^ *P* < 0.05 compared to normal control (G1).

Immunohistochemical evaluation of alpha-smooth muscle actin (*α*-SMA) protein expression in aorta section and histomorphometric analysis from experimental groups are shown in Figure 5. Atherogenic group (G2) showed lesser *α*-SMA staining, compared to normal control group (G1), which had more stain intake of *α*-SMA. On other hand, AV group (G3) and AND HD group (G5) showed strong *α*-SMA protein expression, compared to atherogenic group (G2), while AND LD (G4) showed less *α*-SMA protein expression, compared to AV group (G3) and AND HD group (G5). Atherogenic group (G2) had demonstrated significant reduction (*P* < 0.05) of *α*-SMA protein expression, in comparison to normal control group (G1). However, treated groups (G3–G5) had showed significantly elevated protein expression (*P* < 0.05), compared to atherogenic group (G2).

### 3.7. Western Blot Analysis

**Figure 6:**
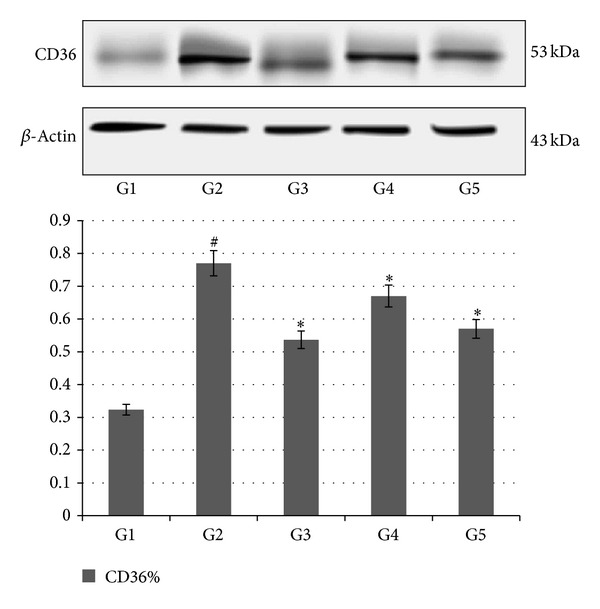
The effect of AND on CD36 expression in aortic tissue homogenate in atherosclerotic rabbits. Group 1: normal control; Group 2: atherogenic control; Group 3: atherogenic + AV; Group 4: atherogenic + AND LD; Group 5: atherogenic + AND HD. The data were analyzed by density color and displayed as mean ± S.E.M. Significance difference was defined as *P < 0.05 compared to atherogenic group (G2); ^#^ *P* < 0.05 compared to normal control (G1).

The effect of AND on CD36 expression in aortic tissue homogenate, in different experimental groups, is shown in Figure 6. Throughout the western blot analysis, we undertook a comparable analysis of CD36 protein in the tissue homogenates of each group, where the *β*-actin protein was considered as the loading control. The result showed that CD36 was significantly inhibited in those rabbits, which received the AV and AND (G3, G4, and G5), compared to the atherogenic group (G2). On the other hand, the expression of CD36 protein was the lowest in AV group (G3), followed by the AND HD group (G5) and then AND LD group (G4). These results had confirmed the role of AND in inhibiting macrophage and lipid accumulation in atherosclerotic rabbits.

## 4. Discussion

MTD was administrated at various doses (50, 100, and 500 mg/kg/day of AND) for four weeks in male and female rabbits. However, AND did not produce any mortality to the rabbits. From this experiment it can be concluded that the rabbits did not have mortality even at maximum dosage of 500 mg/kg body weight. The experimental induction of atherosclerosis, using rabbits, is widely accepted in studies of atherosclerosis. The rabbit's abdominal aorta is a perfect model for atherosclerosis, which can imitate human lesions, if correct food and dietary conditions are given to the rabbit [20, 21]. Using experimental animals, such as rabbits, is crucial, as it allows for consideration of factors, such as diet, length of experiment, pathogenesis of disease, and the impact of dosage, and the duration of feeding, which later yields better and more reliable results. Humans have a specific range of blood lipid profiles, which indicates, whether patients are suffering from various blood and lipid disorders. Unlike rabbits, rabbits do not have specific reference range of blood lipid profiles level for atherosclerotic condition [22]. Therefore, lipid profiles in atherosclerotic rabbits were undertaken, based on a comparison of significant and nonsignificant levels of lipid profile among the five different groups (G1–G5).

This present study had showed that challenging with* Pg* five times a week was sufficient to induce atherosclerosis in rabbits. Furthermore,* Pg* had significantly increased the total cholesterol (TC) level in experimental groups. Physiologically, a high TC level is caused by excessive loading of cholesterol in the liver. These cholesterols are supposed to be metabolized in the liver; however, the excess cholesterol is then recycled in the circulating blood. As the rabbits were in a hypercholesterolemic state, it was noted that AND could significantly reduce the TC level (*P* < 0.05), when compared to the atherogenic group (G2); this had showed that AND has a positive effect in lowering the TC level in a hypercholesterolemic state. Lowering the total cholesterol serum is the primary objective of any treatment in CVD, in attenuating hypercholesterolemic conditions [23]; furthermore, 10% reduction on the concentration of total cholesterol serum can reduce the mortality rate to 15% [24]; total cholesterol could be reduced in several ways, which includes, intake of a sufficient dose, somehow managed to increase LDL receptor proteins in the liver tissue [25–27]. At the same time, there was an increase of LDL receptor mRNA, as the function of receptor is to eliminate LDL in the blood, which further translates into a lower total cholesterol level. The higher LDL level can be attributed to the reduction of LDL receptors by cholesterol in the liver [28]. It is postulated that* Pg* can induce an elevation of TC and LDL levels and reduce HDL levels as well [6]. The reduction in the LDL can be attributed to AND, which has an antihyperlipidemic property [10]. Furthermore, the antioxidant activity of AND might protect LDL against copper mediated modification, by decreasing the binding of Cu^2+^ to LDL. The decreased binding of Cu^2+^ to LDL occurred, when antioxidants reacted with specific amino acid residue on apolipoprotein B, which normally bonds with Cu^2^ [29]. Competition between antioxidants and Cu^2+^ over apolipoprotein B leads to a decreased affinity between apolipoprotein B and Cu^2+^ ions. Moreover, AND can be taken as a supplement to prevent elevation of LDL as this lipoprotein can easily cause health complications (e.g., CVD). The level of HDL in the body indicates a risk factor for the development of atherosclerosis. The lower the HDL level, the higher the risk of having atherosclerosis. Physiologically, HDL transports cholesterol from the peripheral tissue to the liver for catabolism, to be converted into bile salt [30]. This physiological pathway, involving HDL as cholesterol carrier is important in reducing the cholesterol level in the blood. In this study, the rabbits treated with AND and AV had shown increased HDL level in their blood, as against the atherogenic group (G2). This is consistent with clinical studies conducted on simvastatin, which concluded that the latter was able to moderately increase the HDL level [31]. Furthermore, Hernández-Presa et al. [32] have discovered that there was a reduction of plasma TG levels in rabbit, which had been induced with hypercholesterolemia, after treatment with simvastatin; this finding is consistent with our results, which had revealed that AND might reduce the hypercholesterolemia status due to the antioxidant properties of AND.

Vascular inflammation is mediated by numerous cell types that communicate with each other, through a series of cytokine-receptor-mediated interactions, allowing bidirectional crosstalk between resident vascular cells and inflammatory cells [33]. For effective communication to take place, these disparate cell types evolved a common set of soluble cytokine ligands and specific membrane receptors, which allow them to transmit their effects into the cell. Most cytokines initiate a complex and varied repertoire of responses in their target cells and can initiate, advance, and potentially resolve atherogenic inflammation. The microenvironment of the atherosclerotic plaque is a dynamic collection of resident vascular and infiltrating inflammatory cells and their cytokine products. This complex milieu consists of multiple cytokines with redundant, pleiotropic, and opposing effects, and the balance of pro- and anti-inflammatory cytokines often determines plaque severity and stability. Cells present in the lesion must interrogate and respond to this multitude of factors in an appropriate fashion, which together often result in the development and progression of atherosclerosis [34, 35]. The inflammatory process in the atherosclerotic artery may lead to increased blood levels of inflammatory cytokines and other acute-phase reactants [36]. In our study, we had noticed an elevation in the plasma CRP of those rabbits orally challenged with* Pg*. A similar finding was observed by other studies [37]. CRP has thought to be synthesized solely in the liver after stimulation by cytokines, such as IL-6 and TNF-*α* [38]. Previous studies have found that high dietary cholesterol intake can increase the production of atherogenic inflammatory cytokines, such as IL-6 and TNF*α* [39]. In addition, hypercholesterolemia may induce CRP secretions in adipocytes, by reducing the expression of peroxisome proliferator-activated receptor (PPAR-*γ*) [40]. Therefore, administration of AND over 12 weeks could reduce the levels of CRP compared with the atherogenic group (G2), and that could be due to the anti-inflammatory activity of AND. This is consistent with Al-Aubaidy et al. [41], who have concluded that administration of Aliskiren that has antioxidant and anti-inflammatory effects could reduce the TNF*α* and CRP in hyperlipidemic rabbit.

The IL-1 family is composed of four proteins that share sequence homology: IL-1*α*, IL-1*β*, IL1 receptor antagonist, and IL-18. IL-1*β* immune reactivity is found in monocyte, macrophage, EC, and VSMC in human and experimental atherosclerotic plaque. According to several epidemiological and experimental studies, natural or synthetic products, having anti-inflammatory action, are proven to have a strong preventive effect on the development of atherosclerosis [42]. To investigate the potential anti-inflammatory activity of AND as an antiatherogenic agent, we had evaluated the role of AND on the inflammatory progression of early atherosclerosis in rabbits. After 12 weeks of AND treatment along with oral challenge with* Pg*, the levels of IL-1*β* in serum were dropped, due to the treatment of AND. This indicates that AND can prevent atherosclerosis, by alleviating vascular inflammation. This is consistent with Kong et al. [43], who have demonstrated that Kaempferol has an anti-inflammatory effect on early atherosclerosis in rabbits, fed a high cholesterol diets. Furthermore, AV had significantly reduced the level of IL-1*β*, this is due to the fact that AV belongs to the statin category, and statins can act as anti-inflammatory agents as well as having lipid-lowering effects [44]. Emerging data suggest that the proinflammatory cytokine IL-6, secreted by adipocytes, plays an important role in atherosclerosis. In the present study, we have showed that treatment with AV and AND had significantly reduced IL-6. This result has demonstrated that AND has an anti-inflammatory effect in atherosclerosis, which may reduce IL-6 secretion from adipocytes in atherosclerotic rabbits. This finding is in line with that of Zhao and Zhang [45], who have demonstrated that AV has decreased IL-6 secretion in hypercholesterolemic rabbits. Recent studies have demonstrated that atherosclerosis may affect the expressions of a wide variety of adhesion molecules, which increases the production of atherogenic inflammatory cytokines [46]. These studies indicate that* Pg* may induce a systemic inflammation response. We have observed that changes in the circulating concentration of IL-6 were nearly identical to the changes in plasma cholesterol concentration throughout the study, providing further evidence that atherosclerosis might induce systemic inflammatory responses directly.

Macrophages, SMCs, and T cells have been considered as the key cells, involved in atherosclerosis lesion development. Macrophages evolve into foam cells after lipid uptake, which finally leads to early fatty streak formation. T cells, the main cells in cellular immunity, reach intima at an early stage of atherosclerosis formation. Activated T cells produce cytokines, taking part in regulating lesion formation [47]. Meanwhile, as atherosclerosis progresses, vascular SMCs are in proximity to and physically interact with inflammatory cell types, for example, monocytes and macrophages, which play a very important role in further exacerbating the disease [48]. The normal cell structure and morphology of normal healthy aorta can be seen in the normal group (G1). The results showed that foam cells aortic lesion or accumulation of cholesterol deposits was not present in the aortas of normal group (G1), because the rabbits in the normal group were given only a normal diet. In the atherogenic group (G2), the histology results showed that there are thick plaques and lipids accumulation in the tunica intima regions of the aorta. The structure of smooth muscle cells in the media was changed. The intima was irregularly thickened and multilaminated. The internal elastic lamina was also thickened. Fatty streaks were developed at the intimal layer and progressed into plaque. Overall, the plaque in the picture was considered as well as developed atheromatous plaque with a fibrous cap, encircling the top of the plaque. The fibrous cap was composed of proliferating smooth muscle cells, macrophages, lymphocytes, foam cells, and extracellular matrix. Atherogenic group was characterized by an increased population of macrophage-derived foam cells, formed due to increased uptake of oxidized LDL, by smooth muscle cells and macrophages. The formation of foam cells signals the early phase in the development of atherosclerosis [49]. The intimal elastic layer in atherogenic group was not clearly seen between intima and media layer, as the smooth muscle was infiltrated with macrophages and inflammatory cells. It was observed that thickness was prominent in the foam cells in the atherogenic group (G2), compared with the groups treated with AV and AND. This result is consistent with a study conducted on rabbits fed with cholesterol and treated with Nifedipine by Henry and Bentley [50]. The aortic lesions that were present in the groups treated with AV and AND were visibly thinner than those in atherogenic group (G2). Histopathological examination of treated groups revealed a lesser presence of foam cells. Less cholesterol deposits were seen in the aorta of the AV and AND groups, with very little deposits observed in the intima tunica and medica tunica region, and no cholesterol deposit was seen in the adventitia tunica region; this could be due to the anti-inflammatory property of AV and AND. Same phenomenon has been seen in study conducted by Bobek and Galbavý using oyster mushroom in rabbits [51]. Decreased depositions of cholesterol in treated group were represented by lesser lipid laden foam cells, which were originally derived from circulating total cholesterol. The accumulations of atherosclerotic plaques are related to the amount of serum total cholesterol [52]. This was clearly seen when cholesterol found within the arterial wall was proportional to the serum cholesterol level in the lipid profile test. Furthermore, immunohistochemical and western blot tests have confirmed stronger positive expressions of SMCs and reduction of macrophage in aortas of groups, treated with AV and AND. This is consistent with a study conducted by Rosenfeld and Ross on smooth muscle cell proliferation in atherosclerotic lesions in hypercholesterolemic fat-fed rabbits [53].

## 5. Conclusion

In summary, this study had revealed that AND has the ability to improve or inhibit ailments, such as hypercholesterolemia and atherosclerosis, induced in rabbits orally challenged with* Pg*. Furthermore, AND has showed the ability to significantly inhibit the formation and progression of atherosclerosis, induced by* Pg*, which is one of the pathogenic microorganisms in chronic periodontitis. This potential effect could be attributed to the anti-inflammatory effect of AND, which was involved in the reduction of proinflammatory cytokines.

## Supplementary Material

AND was administrated at doses (50, 100 and 500 mg/kg/day) for four weeks in male and female rabbits. No significant difference were recorded in treated groups with AND compared with control group. Also, AND did not cause any mortality throughout the experiment.

## Figures and Tables

**Table 1:** Significance was defined as *P < 0.05 compared to atherogenicgroup (G2). ^#^ *P* < 0.05 indicates significant difference versus normal control (G1).

Animal group	TC (mmol/L)	LDL (mmol/L)	HDL (mmol/L)	TG (mmol/L)
Group 1	1.44 ± 0.01	0.58 ± 0.02	0.57 ± 0.01	0.53 ± 0.01
Group 2	26.21 ± 0.29^#^	24.69 ± 0.14^#^	0.44 ± 0.01^#^	1.31 ± 0.02^#^
Group 3	15.21 ± 0.12*	13.65 ± 0.16*	0.65 ± 0.01*	0.41 ± 0.01*
Group 4	21.22 ± 0.09*	19.17 ± 0.20*	0.61 ± 0.01*	0.61 ± 0.01*
Group 5	16.20 ± 0.18*	14.37 ± 0.22*	0.64 ± 0.02*	0.55 ± 0.01*

TC: total cholesterol; LDL: low-density lipoprotein; HDL: High-density lipoprotein; TG: triglycerides.

Group 1: normal control; Group 2: atherogenic control; Group 3: atherogenic + AV; Group 4: atherogenic + AND LD; Group 5: atherogenic + AND HD.

Statistical analysis of the data was carried out using one-way analysis of variance (ANOVA) and Tukey's post hoc test for average comparison on SPSS 18.0. Mean values ± SEM (*N* = 6) were used. Significance difference was defined as *P < 0.05 compared to atherogenic group (G2); ^#^ *P* < 0.05 compared to normal control (G1).
