# Supplementary material for: Evaluation of the Effect of Andrographolide on Atherosclerotic Rabbits Induced by Porphyromonas gingivalis
Source: Biomed Res Int. 2014 Aug 18;2014:724718. doi: 10.1155/2014/724718 (PMC4151849; doi:10.1155/2014/724718)
Supplement: Supplementary file 1 — AND was administrated at doses (50, 100 and 500 mg/kg/day) for four weeks in male and female rabbits. No significant difference were recorded in treated groups with AND compared with control group. Also, AND did not cause any mortality throughout the experiment. [file 724718.f1.pdf]

Maximum Tolerated Dose (MTD) of AND on rabbits

Table S1: Maximum tolerated dose of AND based on body weight effect of male rabbit.

Animal groups	Duration of treatment (Week)					Weight Gain/Loss (g)
	0	1	2	3	4	
**Control**	3040	3080	3130	3230	3395	355 ± 63.522
**AND (50 mg/kg)**	3110	3255	3330	3375	3450	340 ± 57.909
**AND (100 mg/kg)**	3100	3180	3235	3300	3365	265 ± 46.027
**AND (500 mg/kg)**	3155	3180	3250	3315	3390	235 ± 43.261

Values are expressed as mean ± S.E.M. No statistical difference between control and AND treated group ($p<0.05$).

Table S2: Maximum tolerated dose of AND based on body weight effect of female rabbit.

Animal groups	Duration of treatment (Week)					Weight Gain/Loss (g)
	0	1	2	3	4	
**Control**	3115	3170	3245	3360	3435	320 ± 59.097
**AND (50 mg/kg)**	3205	3255	3330	3455	3500	295 ± 56.555
**AND (100 mg/kg)**	3140	3235	3380	3440	3505	365 ± 67.026
**AND (500 mg/kg)**	3105	3125	3205	3315	3476	371 ± 68.506

Values are expressed as mean ± S.E.M. No statistical difference between control and AND treated group ($p<0.05$).
